# News and views

**DOI:** 10.1111/iwj.13953

**Published:** 2022-09-21

## 1 NORTHWESTERN UNIVERSITY

### 1.1 Scientists identify key mechanism controlling skin regeneration

#### 1.1.1 Early molecular switch inside nucleus is crucial for deciding fate of skin stem cells

It is sunburn season. Many of us have experienced the pain and peeling that comes from the unprotected time in the sun, but we may not focus on a remarkable and vital part of the process: the regeneration of skin as the damaged tissue is replaced with new.

Even without sunburn, the outer layer of skin, the epidermis, is constantly turning over to replace dead or damaged cells throughout our lifetime. This epidermal layer provides an essential barrier for the human body, reducing water loss and combating environmental threats. Scientists are working to identify the molecular mechanisms controlling skin epidermal regeneration, but much remains poorly understood.

Now a Northwestern University research team has identified a molecular switch, through a protein called CDK9, that plays an early and critical role in the skin stem cell differentiation process. This switch is ‘off’ in the stem cells. When the switch is turned on, a specific group of genes is immediately activated to trigger downstream gene regulators, allowing the skin cells to progressively gain barrier function. The findings have relevance for improved understanding of cancer and wound healing, in addition to the fundamental understanding of skin regeneration.

Bao is an assistant professor of molecular biosciences in the Weinberg College of Arts and Sciences and an assistant professor of dermatology at Northwestern University Feinberg School of Medicine. Her lab studies the fundamental biology of the process of skin stem cell differentiation.

#### 1.1.2 Discovery of the switch

The integrity of the skin epidermis relies on subsets of skin stem cells to continuously self‐renew or differentiate, compensating for daily wear and tear. The differentiation process involves significant changes from more than 6000 genes, ceasing stem cell proliferation while activating barrier‐function genes.

Integrating genomics, genetics and pharmacological inhibition to human skin models, Bao and her team identified that the kinase activity switch of the protein CDK9 plays a key role in the decision of cells to initiate differentiation and progressively acquire the barrier function of the tissue. The kinase activity is off in the stem cell state, and the rapid‐response genes directly controlled by the kinase are suppressed. When the kinase activity is on, the rapid‐response genes are activated, which subsequently induce the downstream effectors, a group of transcription factors that can further drive the expression of barrier‐function genes.

CDK9 (cyclin‐dependent kinase 9) plays crucial roles in modulating gene expression at the step of ‘transcription’, a process of copying specific DNA regions to RNA, before RNA can serve as templates for synthesising new proteins. In the stem cell state, CDK9 is maintained in the ‘off’ state when bound together with the proteins AFF1 and HEXIM1 on DNA, awaiting specific cellular signals such as the activation of protein kinase C signalling. Once the signalling is activated, this is sufficient to switch CDK9 from the inactive to the active state, allowing the rapid synthesis of RNA from the genomic regions directly bound by CDK9, the researchers found.

The switch is a quick one. ‘All the components are poised for action deep inside the stem cells’, Bao said. When the stem cell receives specific external signals, the response inside the nucleus is very fast, with activated CDK9 quickly causing rapid‐response genes such as ATF3 to be expressed within as short as 1 h. The expression of ATF3 potently induces several downstream transcription factors to rewire the cell fate towards differentiation. This quick switch for gene activation is also built upon the pre‐recruitment of RNA‐synthesis machinery together with CDK9 to the rapid‐response genes, before the signalling is activated.

‘We are probing the unknown’, Bao said. ‘Stem cell regulation is fundamental for sustaining the integrity of human tissue. We have found a key mechanism initiating the fate switch of skin stem cell towards differentiation, an integral process of regeneration. Learning more about the fundamental molecular mechanisms can help in the understanding of many different human diseases’.

## 2 3M

### 2.1 3M upgrades make negative pressure wound therapy with instillation easier for clinicians to initiate

3M Health Care's Medical Solutions Division has advanced the delivery of 3M Veraflo Therapy. A new 3M Veraflo Cleanse Choice Complete™ Dressing Kit and a software upgrade for the 3M V.A.C. ULTA Therapy Unit was introduced at no increased cost to the customer. These new offerings help simplify the care delivery processes for clinicians using Veraflo Therapy (negative pressure wound therapy with instillation) and help make dressing changes easier, faster and less painful for their patients as compared to previous Veraflo Therapy dressings.

Wound care experts use Veraflo Therapy for patients with open soft tissue wounds to help cleanse and stimulate formation of granulation tissue. A recent publication by Gabriel et al., ‘Effects of Negative‐Pressure Wound Therapy with Instillation versus Standard of Care in Multiple Wound Types: Systematic Literature Review and Meta‐Analysis’, shows that Veraflo Therapy demonstrated significant clinical advantages and economic savings vs other advanced wound therapies, including traditional negative pressure wound therapy (NPWT). When Veraflo Cleanse Choice Complete Dressing is used in conjunction with Veraflo Therapy, the combination helps to disrupt, soften, solubilise and remove thick, exudate and non‐viable tissue when immediate wound cleansing is needed or when surgical debridement must be delayed or is not possible.

‘3M offers a broad range of wound care solutions because clinicians need a robust tool selection’, said Ronald Silverman, MD, 3M Health Care Senior Vice President of Clinical Affairs and Chief Medical Officer. ‘We continue to find ways to innovate to meet their unique wound care needs and address the changing nature of wound care. Today's wound care patients are often sicker and have more comorbidities, making their wounds more complex to treat and increasing the demands on clinicians' time. We strive to make our products easier to use to help save clinicians' valuable time and, ultimately, transform patient outcomes and improve their lives’.

#### 2.1.1 Software upgrade

The V.A.C.® Ulta Therapy Unit is the only negative wound pressure therapy device that delivers four therapy options, enabling clinicians in the acute care setting to manage open abdomens, closed incisions and open soft tissue wounds across a wide range of complexities, including size, contamination and aetiology.

The device's newly upgraded software, which includes the 3M Smart Instill Feature for use with Veraflo Therapy, automates therapy initiation and makes it easier for clinicians to initiate therapy. Additional upgraded functionalities include animated video troubleshooting, an instil phase postpone feature, a therapy inactive alarm time delay providing clinicians a choice to delay alarms during dressing changes, along with the updated default therapy settings. These new default settings are based on the recommendations of a global panel of wound care leaders as outlined in a 2020 NPWT with instillation consensus guidelines update document published in the *International Wound Journal*. Kim PJ, Attinger CE, Constantine, T et al. Int Wound J.2020;17:174–186.

‘Veraflo Therapy with Smart Instill Feature helps remove the guesswork and difficult aspects of setting up NPWT with instillation for patients. You push a button or two and the software guides you through the decision‐making process, significantly decreasing the time involved in administering the therapy’, said Marc Matthews, MD, MS, MCG, FACS, Arizona Burn Center, Associate Director.

The software upgrade has been completed in all US rental units. Owned units will be upgraded beginning in 3Q22.

#### 2.1.2 Dressing kit

Based on clinician feedback, the new Veraflo Choice Cleanse Complete Dressing Kit was developed to simplify dressing application and reduce the number of SKUs and ancillary products used to manage open soft tissue wounds. The kit's new dressing is a single piece, which users may find easier to apply than the current three‐piece dressing. The three‐piece dressing is still an available option for users who are accustomed to this design. This kit is also the first time the 3M Dermatac Drape is included in a Veraflo Therapy Dressing Kit. Dermatac Drape is constructed of a unique silicone‐acrylic hybrid material that is gentler than acrylic drapes on patients' skin at removal, leading to less painful dressing changes. During application, the drape can also be repositioned, which simplifies clinicians' efforts.

The combination of the Smart Instill Feature and the Veraflo Cleanse Choice Complete Dressing Kit makes Veraflo Therapy easier than ever to apply. Now, clinicians can choose a more innovative therapy option, without the challenge of increased complexities or costs while positively impacting the patient's experience. For more information, visit www.3M.com/Veraflo.

## 3 ORGANOGENESIS

### 3.1 Organogenesis receives FDA clearance for next generation PuraPly surgical solution

Organogenesis, a leading regenerative medicine company, has received US Food and Drug Administration (FDA) 510k Clearance for PuraPly MZ, a brand extension to the PuraPly product portfolio. PuraPly MZ leverages the innovative properties of our PuraPly technology engineered into a micronized (powdered) form to provide surgeons with an option for complex surgical wounds.

‘Securing FDA Clearance for PuraPly MZ is a significant milestone for the company as we continue to expand into the surgical and sports medicine market’, said Gary S. Gillheeney, Sr. President and Chief Executive Officer of Organogenesis. ‘We are very pleased to provide surgeons addressing complex surgical wounds with access to this extension of our widely used PuraPly technology. This new solution demonstrates Organogenesis's commitment to delivering innovative advanced technologies that improve surgical outcomes and patient experience’.

#### 3.1.1 PuraPly product portfolio

Of the 40 to 50 million surgeries in the United States each year, up to 28% of surgical sites must be left open to heal. To support clinicians and patients with these complex post‐surgical wounds, Organogenesis developed PuraPly MZ, a powder that is designed to support wound healing in deep, tunnelling and complex wounds. PuraPly MZ consists of native structured collagen that is designed to allow for maximum coverage and maintenance of optimal contact with the contours of the wound surface to support healing.

Organogenesis offers a variety of solutions for the advanced wound care and surgical and sports medicine markets. For information on all Organogenesis's wound care solutions, visit Organogenesis.com.

